# The comparative efficacy and safety of anti-CD20 monoclonal antibodies for relapsing-remitting multiple sclerosis: A network meta-analysis

**DOI:** 10.1016/j.ibneur.2021.08.003

**Published:** 2021-08-27

**Authors:** Mohammad Z.I. Asha, Yousef Al-Asaad, Sundos F.H. Khalil

**Affiliations:** a Alfardan Medical with Northwestern Medicine, Doha, Qatar; b Gharrafat Al Rayyan Health Center, Doha, Qatar; c Jordan University, Amman, Jordan

**Keywords:** Monoclonal antibody, Multiple sclerosis, Ocrelizumab, Relapsing-remitting multiple sclerosis, Network meta-analysis

## Abstract

With the recent successful targeting of B lymphocytes in patients with multiple sclerosis (MS), treatment with anti-CD20 monoclonal antibodies (mAbs) may represent a promising managemental approach, particularly for those with relapsing/remitting MS (RRMS). A network meta-analysis was conducted based on a comprehensive search in Embase, PubMed, and the Cochrane Library to assess the comparative efficacy and safety of currently available anti-CD20 monoclonal antibodies (mAbs), including rituximab, ocrelizumab, and ofatumumab, versus a common comparator (interferon beta-1a [INFβ-1a]) in RRMS patients recruited in randomized clinical trials (RCTs). In a frequentist network meta-analytical model, annualized relapse rates (ARRs) and safety outcomes were expressed as risk ratios (RRs), whereas relapse-free events were expressed as odds ratios (ORs). Treatment ranking was performed using P-scores. The certainty of evidence was appraised using the GRADE approach. Five publications reported the outcomes of seven RCTs (3938 patients, 67.09% females). Compared to INFβ-1a, ocrelizumab reduced the risk of ARR (RR = 0.56, 95% CI, 0.50–0.64), serious adverse events (RR = 0.17, 95% CI, 0.09–0.30), and treatment discontinuation due to adverse events (SAEs, RR = 0.60, 95% CI, 0.39–0.93), and it was associated with higher odds of no relapses (OR = 2.47, 95% CI, 2.00–3.05). Ocrelizumab ranked best among all other treatments in terms of reducing ARR and SAEs. The quality of evidence was low for ocrelizumab, low to moderate for rituximab, and high for ofatumumab. Further large-sized, well-designed RCTs are needed to corroborate the efficacy and safety of ocrelizumab and other anti-CD20 mAbs in RRMS.

## Highlights

• B cell depletion via the anti-CD20 therapy is promising in relapsing-remitting MS.
• Data is currently available for ocrelizumab, rituximab, and ofatumumab.
• Ocrelizumab was the most efficacious biological agent in reducing relapse.
• Ocrelizumab reduced also the risk of treatment discontinuation due to adverse events.

## 1 Introduction

Multiple sclerosis (MS) is an autoimmune, inflammatory disease of the central nervous system (CNS) characterized by neurological defects and physical and cognitive disabilities associated with varying degrees of myelinic and axonal destruction. In 2016, the disease was prevalent among more than 2.22 million patients worldwide, which represents a 10.4% increase in the age-standardized prevalence compared to 1990 (Wallin et al., 2019). In general, the disease has variable and unpredictable courses. Relapsing remitting MS (RRMS) is the most common form (85% of MS patients), where the patients experience acute attacks followed by periods of remissions. Other forms include primary progressive MS (gradual worsening without relapses or remissions), secondary progressive (steady worsening after RRMS), and progressive-relapsing MS (progressive with intermittent periods of worsening symptoms) (Ghasemi et al., 2017). The etiopathogenic mechanisms of MS remain elusive; of them, immune system factors that mediate CNS damage (Coyle, 2020) have been particularly prominent in RRMS. Traditionally, the immunoreactive concepts of MS were based on CD4 + T cells sensitized to the myelin components of CNS (Kuchroo and Korn, 2013). A number of broad-spectrum immunomodulatory drugs, such as teriflunomide, natalizumab, interferon-β, and fingolimod. Unfortunately, a number of these medications are often criticized due to their inferior safety profiles; they might be associated with flu-like symptoms, cancer, and serious opportunistic infections (Dendrou et al., 2015).

Contemporaneously, there has been a growing body of evidence indicating the involvement of B cells in MS pathophysiology (Coyle, 2020). This was supported by the existence of oligoclonal bands (OCBs) and the production of intrathecal immunoglobulins in the cerebrospinal fluid of MS patients (Tomescu-Baciu et al., 2019, Deisenhammer et al., 2019), as well as the detection of myelin-specific antibodies that mediate complement-dependent oligodendrocyte loss and demyelination in active MS lesions (Liu et al., 2017). Interestingly, the most impressive data comes from the promising outcomes of anti-CD20 monoclonal antibodies (mAbs) in RRMS. Based on the accumulating evidence, the US Food and Drug Administration has recently approved the humanized anti-CD20 monoclonal antibody ocrelizumab (OCR) for RRMS (Food and Drug Administration, 2017). Additionally, other anti-CD20 mAbs, such as rituximab (RTX), ofatumumab (OFA), and ublituximab (UTX), are currently under investigation in phase II and III trials. However, head-to-head trials of these medications in RRMS are not yet available; thus, the comparative performance of their efficacy and safety could not be effectively concluded. Besides, the published collective reviews up to date have relied on a qualitative synthesis of available trials or a quantitative analysis of all disease modifying agents. In the present article, we sought to perform a systematic review and network meta-analysis to investigate the comparative clinical efficacy and safety of anti-CD20 mAbs based on the reported data from randomized clinical trials (RCTs) in patients with RRMS to provide deep insights into the most efficacious and safe anti-CD20 medication to reduce relapses.

## 2 Methods

The present article was formulated based on the guidelines implied by the Preferred Reporting Items for Systematic reviews and Meta-analyses (PRISMA) extension statement for systematic reviews and network meta-analyses (Hutton et al., 2015).

### 2.1 Search strategy and selection criteria

Two independent authors searched Embase, PubMed, and the Cochrane Library database until August 20, 2020. There were no date limits regarding the publication date of the included studies. The strategy was based on specific keywords and Boolean operators as demonstrated in Appendix 1. The protocol of the current article was not registered.

All RCTs which have investigated the efficacy and safety of RTX, OCR, OFA, or UTX in at least one arm of RRMS patients were eligible. The used medications should have been used as monotherapy, and the control arm could be other mAbs, interferon beta-1a (INFβ-1a), or placebo. Studies written in English language and peer-reviewed articles were only included. Analytic studies which included subsets of patients from previously published trials were excluded. Phase I (open-label) trials, retrospective studies, meta-analyses, conference abstracts, and review articles were not eligible.

### 2.2 Types of outcomes measures

Clinical efficacy outcomes included the annualized relapse rate (ARR) in the intention-to-treat cohorts, where applicable. The primary safety outcome was the number of patients with serious adverse events (SAEs). Secondary outcomes included the proportion of relapse-free patients at 24 weeks, as well as the proportion of patients with any adverse event (AE) and the proportion of those who had discontinued treatment due to AEs.

### 2.3 Study selection and data extraction

All obtained records were screened independently by two authors. The results were entered in a reference organizing software (Endnote version X9), where duplicate records were identified and deleted. The titles and abstracts of all records were screened based on the eligibility criteria, and the full-text version of potentially eligible studies was checked for the primary and secondary outcomes. Any disagreement between authors regarding eligible studies was resolved by discussion. Subsequently, data was extracted into a specific spreadsheet (Microsoft Excel 2016). The following data was collected: 1) study-related data: authors, date of publication, trial name, trial phase, follow-up period, and the location; 2) cohort-related data: the mean age of the total cohort, gender, and the mean scores of the Expanded Disability Status Scale (EDSS); 3) intervention-related data: study arms, type of the medication, regimen, and the number of patients in each arm; 4) outcome data: ARR, the proportion of patients with no relapse at 24 weeks, as well as the proportion of any adverse event, SAEs, and patients who had discontinued treatments due to AEs.

For active treatments, data was collected for patients who had received the following regimens: RTX 1000 mg (IV infusion on days 1 and 15), OCR (two 300-mg IV infusions within two weeks followed by subsequent 600-mg infusions every 6 months), and OFA (monthly 20 or 30 mg subcutaneous injections). The regimens of OCR and OFA were selected based on the FDA approval schemes (Ligi et al., 2020, The U.S. Food and Drug Administration, 2017), whereas the RTX regimen was included based its off-label use for RRMS in multiple countries worldwide (Ineichen et al., 2020). The outcomes of the two doses of subcutaneous OFA (20 mg and 30 mg) were merged together into a single node to avoid network disconnection and to increase the statistical power of such an outcome (Chaimani et al., 2019, Ter Veer et al., 2019).

### 2.4 Risk of bias

The methodological quality of RCTs was assessed via the revised version of the Cochrane’s Risk of Bias Tool, namely RoB 2 (Sterne et al., 2019). Two independent authors provided their judgements based on answering the signaling questions of the RoB2 manual; these responses were related to five major domains, including the randomization process (Domain 1 [D1]), deviations from intended interventions (D2), missing outcome data (D3), measurement of the outcome (D4), and selection of the reported results (D5). The answers to the signaling questions were provided as Yes “Y”, Probably Yes “PY”, Probably No “PN”, No “N”, or No Information “NI”.

### 2.5 Statistical analysis

During data collection, missing mean values and their corresponding standard deviations were computed by combining the means and SDs of patients’ subgroups as described in the Cochrane Handbook for Systematic Reviews of Interventions.(Higgins et al., 2019) Subsequently, pairwise meta-analyses were carried out by calculating risk ratios (RRs) and their respective 95% confidence intervals (95% CIs) for ARR and safety outcomes, while odds ratios (ORs) and 95% CIs were used to express the results of relapse-free events. The network meta-analysis was then constructed in a frequentist framework using the *netmeta* library in R software (R i386 version 4.0.0), (Harrer et al., 2019) and the biological treatments were entered as active treatments, and INFβ-1a was the common comparator. Heterogeneity testing (within designs) was performed using a *I*^*2*^ test, with a substantial heterogeneity at *I*^*2*^ > 50%. A fixed-effects model was applied in the instance of low heterogeneity; otherwise, a random-effects model was adopted. Assessment of inconsistency (the difference between direct and indirect evidence) was performed locally using net splitting (back‐calculation) for eligible comparisons (whenever available) and globally using a design-by-treatment interaction approach (Higgins et al., 2012). Transitivity was assessed via evaluating the distribution of potential effect modifiers across trials, (Salanti et al., 2014) including age, time since symptom onset, time since diagnosis, EDSS score, and the number of relapses in the past year. League tables were used to present all possible pairwise comparisons in off-diagonal cells (Chaimani and Salanti, 2015). Additionally, network forest plots were produced to visualize the effect estimates of drugs as compared to the common comparator. Treatment ranking was expressed as P-scores, which represent the frequentist alternative of the Surface Under the Cumulative Ranking curve (SUCRA) (Rücker and Schwarzer, 2015). P-scores ranged between 0 and 1; higher treatment ranks (close to 1) indicated that the drug is certain to perform the best (caused low risk of ARR, low risks of safety outcomes, and high odds of relapse-free events). Assessment of publication bias via comparison-adjusted funnel plots was not possible owing to the small number of studies included within each pairwise comparison (Chaimani and Salanti, 2015). Statistical significance was considered at a two-tailed p value of <0.05.

### 2.6 Certainty of evidence

The certainty of evidence was assessed using the Grading of Recommendations Assessment, Development and Evaluation (GRADE) for network meta-analyses (Schünemann, 2019). Given that all the eligible studies were RCTs, evidence rating started at the highest level of certainty “high” as per official recommendations.(Schünemann, 2019). Each trial was then rated down based on the results of assessment of risk of bias (RoB2), inconsistency, imprecision, and indirectness. Publication bias was not included in the GRADE analysis since it was not assessed statistically in our analysis.

## 3 Results

### 3.1 Results of the search process

**Fig. 1:**
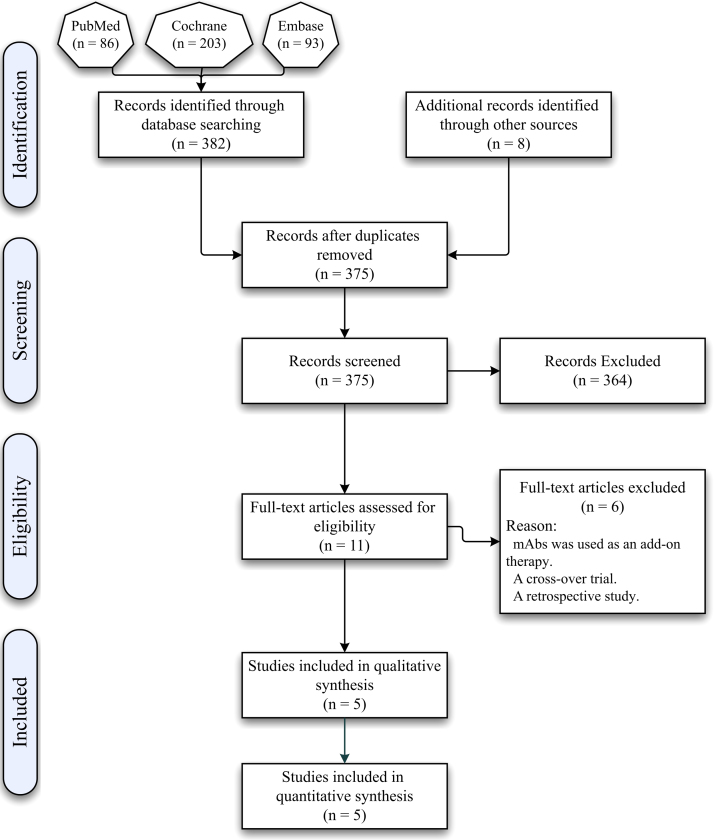
A PRISMA flowchart showing the search process used in the current review.

The total number of obtained records was 390, including eight records identified from the bibliographies of screened articles. After the exclusion of duplicate records (n = 15), 11 articles met the eligibility criteria, and the full-text versions of such articles were downloaded. However, six articles were excluded due to the following reasons: including active mAbs as an add-on treatment (Honce et al., 2019, Naismith et al., 2010, Cross et al., 2012), implementing a cross-over design (Fox et al., 2021, Sorensen et al., 2014), and collecting data in a retrospective manner (Bellinvia et al., 2020). Therefore, ultimately, five RCTs were included in the meta-analysis (Fig. 1).

### 3.2 Characteristics of the Included Studies

As demonstrated in Table 1, a total of 3938 patients with RRMS were recruited in the eligible trials (67.09% females). The mean age of the participants ranged between 37.0 and 40.2 years. Two separate articles contained published data for two trials in each (Hauser et al., 2017). In addition, three trials were two-arm studies (Hauser et al., 2017, Hauser et al., 2008, Hauser et al., 2020), and two articles were three-arm studies (Bar-Or et al., 2018, Kappos et al., 2011). No eligible trials included UTX for RRMS patients.

**Table 1:** Characteristics of the included studies.

**Authors and study name**	**Conducted in**	**Design**	**Age (mean ± SD)**	**T (M/F)**	**EDSS (mean ± SD)**	**follow-up time (weeks)**	
Hauser et al. (2017) (OPERA I)	141 centers across 32 countries	Phase III, double-blind	**37 ± 9.2**	821 (279/542)	2.8 ± 1.3	96	**First Arm:** Ocrelizumab 600 mg (First cycle: two 300-mg IV infusions on days 1 and 15. Subsequent cycles: a single 600-mg infusion) **Second Arm:** Interferon beta-1a 44 μg (S/C three times weekly)
Hauser et al. (2017) (OPERA II)	166 centers across 24 countries	Phase III, double-blind	37.3 ± 9.1	835 (284/551)	2.81 ± 1.3	96	**First Arm:** Ocrelizumab 600 mg (Two 300-mg IV infusions on days 1 and 15 followed by a single 600-mg infusion) **Second Arm:** Interferon beta-1a 44 μg (S/C three times weekly)
Hauser et al. (2008)	32 centers in the United States and Canada	Phase II, double-blind	40.23 ± 8.6	104 (23/81)	2.5 ± 1.08	48	**First Arm:** Rituximab 1000 mg (IV infusion on days 1 and 15) **Second Arm:** Placebo (IV infusion on days 1 and 15)
Kappos et al. (2011)	79 centers across 20 countries	Phase II, double-blind	37.22 ± 8.9	163 (60/103)	3.26 ± 1.47	48	**First Arm:** Ocrelizumab 600 mg (First cycle: two 300-mg IV infusions on days 1 and 15. Subsequent cycles: a single 600-mg infusion) **Second Arm:** Interferon beta-1a (IM once a week for 24 weeks) **Third Arm:** Placebo
Hauser et al. (2020) (ASCLEPIOS I)	385 sites in 37 countries	Phase III, double-blind	38.35 ± 8.91	927 (292/635)	2.96 ± 1.36	96	**First Arm:** Ofatumumab 20 mg (S/C injections every 4 weeks for 30 months **Second Arm:** Teriflunomide 14 mg (For 30 months: teriflunomide once daily + S/C placebo)
Hauser et al. (2020) (ASCLEPIOS II)		Phase III, double-blind	38.09 ± 9.4	955 (317/638)	2.88 ± 1.35	96	**First Arm:** Ofatumumab 20 mg (S/C injections every 4 weeks for 30 months) **Second Arm:** Teriflunomide 14 mg (For 30 months: teriflunomide once daily + S/C placebo)
Bar-Or et al. (2018) (MIRROR)	10 countries	Phase IIb, double-blind	37.47 ± 9.54	133 (41/92)	Non-available	24	**First Arm:** Ofatumumab 30 mg (S/C injections every 12 wk) **Second Arm:** Placebo

### 3.3 Network structure

**Fig. 2:**
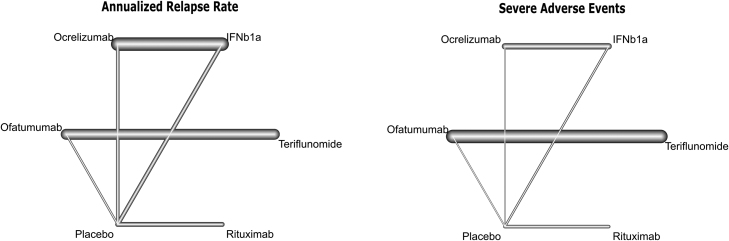
Network maps of eligible comparisons of the primary outcomes of efficacy (annualized relapse rate) and safety (serious adverse events). The thickness of lines represents the number of studies in each comparison.

Fig. 2 depicts the network structure of anti-CD20 mAbs and their comparator arms for the primary outcomes. Interventions entailed OCR, OFA, RTX, interferon beta-1a (IFN β-1a), teriflunomide, and a placebo intervention. Eligible trials included comparisons of active medications versus a placebo intervention, except one trial (Hauser et al., 2017) which has compared OCR and IFN β-1a. Primary outcomes were reported in all trials (ARR in 3897 and SAEs in 3895 patients).

### 3.4 Risk of bias and certainty of evidence

The results of authors’ judgements regarding the risk of bias in the included trials are demonstrated in Appendix 2 and Appendix 3. The method and appropriateness of randomization were not explicitly mentioned in two trials; (Hauser et al., 2008, Hauser et al., 2020) thus, the relevant items in D1 were judged as “PY”. Focusing on the study of Hauser (2008), although baseline characteristics of patients were balanced, the percentage of patients with gadolinium-enhancing lesions was significantly lower in the active treatment group (RTX) than in the placebo group. This might indicate problems in the randomization process, which might have led to bias in the intervention effect estimate. Additionally, the rates of dropouts over 48 weeks were relatively high in the active treatment arm (23.9%) and very high in the placebo arm (40%), and there was an imbalance in the causes of discontinuation due to AEs between the study arms (Hauser et al., 2008). Such factors might have contributed to attrition bias. In the study of Kappos (2011), treatment assignment was masked for patients during the trial. However, in the IFNba1 group, raters were only blinded to allocation, but not other subsequent procedures (D2 was judged as “high risk”). Additionally, an intention-to-treat (ITT) analysis was not performed on the ARR outcome; yet, it was applied on relapse-free events and on safety outcomes (D4 was judged as “high risk”) (Kappos et al., 2011). Finally, financial conflicts of interest were reported in two trials, (Hauser et al., 2008, Kappos et al., 2011) and these conflicts might have caused serious concerns in allocation concealment in the study of Hauser et al. (2008) possibly to produce intervention groups which are imbalanced in favor of the active intervention.

Regarding the grading of quality of evidence, the confidence in the estimated effects were generally low for OCR due to methodological limitations in study designs, low to moderate for RTX due to limitations in the study designs and imprecision, and high for OFA. Detailed assessment results are demonstrated in Appendix 4.

### 3.5 Testing for heterogeneity and inconsistency

Heterogeneity testing indicated that the *I*^*2*^ value was consistently low for all the outcomes (the *I*^*2*^ values ranged between 0% and 22.4%, Appendix 5). The inconsistency with an assumed full design-by-treatment interaction was non-significant (Q values ranged between 0.24 and 1.98, p > 0.05, Appendix 5), indicating a lack of inconsistency. Assessment of the transitivity assumption showed that the potential effect modifiers were similarly distributed across studies (Appendix 6). Furthermore, there were no comparisons with a significant disagreement between direct and indirect evidence as revealed by the net splitting analyses (back-calculation, Appendix 7). Based on these findings, fixed-effects meta-analysis models were applied for all the networks.

### 3.6 Primary efficacy and safety outcomes

The main results of the primary efficacy and safety outcomes are demonstrated in a league table (Table 2). The included trials showed that the treatment with biological agents was associated with lower ARRs compared to other non-biological arms in distinct pairwise comparisons. That is, the rate of ARR was significantly lower with OCR compared to three comparator arms: INF β-1a (RR = 0.56, 95% CI, 0.50–0.64), placebo (RR = 0.36, 95% CI, 0.26–0.51), and teriflunomide (RR = 0.33, 95% CI, 0.14–0.80). Additionally, the rate of ARR decreased with OFA compared to teriflunomide (RR = 0.45, 95% CI, 0.38–0.52) and RTX compared to placebo (RR = 0.56, 95% CI, 0.40–0.81). Based on P-scores, OCR and OFA were ranked best on reducing the risk of ARRs among all treatments (Appendix 8.1).

**Table 2:** Network meta-analysis of the primary efficacy and safety outcomes.

*Results are expressed as RR (95% CI). Comparisons between the column-defining and row-defining interventions should be read from left to right. The outcomes in bold and underline are statistically significant results.  primary efficacy outcome (annualized relapse rate);  primary safety outcome (the proportion of patients with serious adverse events); Treatment.

**Fig. 3:**
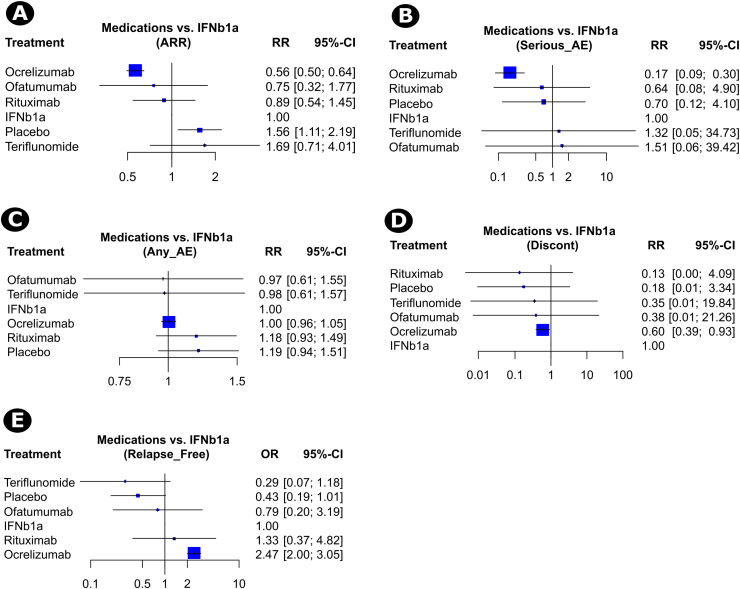
Forest plots of the network meta-analysis of the efficacy and safety of anti-CD20 mAbs, including the risk of developing annualized relapse rate (A), serious adverse events (B), any adverse event (C), and discontinuation of treatments due to adverse events (D), as well as the odds of relapse-free events (E).

Regarding safety, SAEs had generally occurred in 6.73% (262 out of 3895 patients). The incidence of SAEs was similar across anti-CD20 mAbs. However, OCR was associated with a lower risk of SAEs compared to INF β-1a (RR = 0.17, 95% CI, 0.09–0.30). The superior efficacy and safety of OCR over INF β-1a was confirmed in the respective forest plots (Fig. 3A and B). Of note, OCR was ranked best on reducing the risk of SAEs (Appendix 8.2).

### 3.7 Secondary outcomes

The outcomes of the frequency of relapse-free events (efficacy) as well as treatment discontinuation due to adverse events and the incidence of all adverse events (safety) are demonstrated in Appendix 9. OCR was associated with higher odds of no relapses at week 24 compared to INF β-1a (OR = 2.47, 95% CI, 2.00–3.05), placebo (OR = 5.71, 95% CI, 2.42–13.46), and teriflunomide (OR = 8.52, 95% CI, 2.08–34.94). Besides, a higher proportion of patients experienced no relapse after receiving RTX compared to placebo (OR = 3.08, 95% CI, 1.17–8.10) and teriflunomide (OR = 4.59, 95% CI, 1.04–20.20) and OFA compared to teriflunomide (OR = 2.74, 95% CI, 2.23–3.73, Appendix 9.1). Regarding safety, there was no difference in the risk of developing adverse events across different medications (Appendix 9.2). Nonetheless, the risk of treatment discontinuation due to adverse events was significantly lower in the OCR arms compared to INF β-1a (RR = 0.60, 95% CI, 0.39–0.93, Appendix 9.3). Detailed values of P-scores for the treatment rankings regarding secondary efficacy and safety outcomes indicated that OCR and RTX performed best on increasing the odds of relapse-free events (Appendix 8.3), OFA, IFNb1a, and OCR on reducing the risk of any AE (Appendix 8.4), and RTX on reducing the risk of discontinuation of treatments due to AEs (Appendix 8.5).

## 4 Discussion

The outcomes of neuroimmunological studies have provided important insights into the pathogenesis of multiple sclerosis and have inspired the researchers to test targeted immune therapies on patients with different forms of the disease. The pathogenic role of B cells is evident from recent RCTs, and therapeutic targeting of these cell lineages has induced promising outcomes. In the present study, we sought to compare the efficacy and safety of the novel anti-CD20 agents. Results showed that OCR, OFA, and RTX had the highest probability of being better than other non-mAbs interventions in terms of reducing the ARR. Additionally, only OCR was associated with lower rates of SAEs compared to other non-biological agents. However, no significant differences were identified between the three biological drugs in terms of efficacy and safety outcomes.

These findings were relatively similar to a recent network meta-analysis. In their study, Samjoo et al. (2020) sought to compare the efficacy and safety of OFA to other disease-modifying agents on patients with relapsing MS. The authors found significant improvements in disability progression and ARR in treatment arms using OFA (subcutaneously) based on the outcomes of 34 trials; yet, however, these effects were comparable to other mAbs, such as natalizumab, alemtuzumab, and OCR. Other similar meta-analyses have also revealed promising results for OCR, natalizumab, and alemtuzumab with no significant differences between different biological agents (Mccool et al., 2019; Lucchetta et al., 2018; Fogarty et al., 2016). These results support the growing trend of B cell depletion via the anti-CD20 therapy to alleviate the burden of relapses in RRMS. CD20 is a four-transmembrane surface molecule expressed on the surface of subpopulation of B cells throughout their maturation, including pre-B cells, mature B cells, and memory cells, but not plasmablasts and antibody-producing plasma cells (Payandeh et al., 2019). Trials to date have investigated RTX and its humanized successors OCR and OFA, which act by targeting B cells through cellular cytotoxicity, complement-dependent cytotoxicity, and induction of apoptosis. The significant effects of anti-CD20 mAbs on relapses may be partly explained by the ability of these medications to suspend a subset of B lymphocytes which are pathogenic in MS patients. Indeed, B cells are potent antigen presenting cells which mediate T cell activation, and they can also produce pathogenic antibodies (Comi et al., 2020). Moreover, a subset of B cells are active producers of cytokines that regulate the proinflammatory (interleukin [IL]−6) as well as the anti-inflammatory responses (IL-10) (Lehmann-Horn et al., 2013). In an experimental autoimmune encephalomyelitis mice model, Barr et al. (2012) indicated that B cell depletion has improved autoimmune disease progression by inhibiting IL-6-producing B cells. Therefore, it seems that B cell therapy may regulate specific key traits of B cells, such as IL-derived inflammatory responses and antigen presentation. However, there are many aspects in the pathophysiological pathway which remain elusive. For instance, future in-vitro and in-vivo studies are required to identify the factors that could drive B cells into the CNS, the pathways through which they travel, and whether the cell traffic is transient or persistent. Additionally, it is necessary to identify other CNS niches in which B cell populations might thrive.

In agreement with the significant role of anti-CD20 agents in B cell depletion, RTX has proven effective in RRMS; however, the available evidence is primarily based on non-randomized studies of off-label RTX infusion (Yamout et al., 2018, Airas et al., 2020). This is because RTX has only approved for the treatment of non-Hodgkin’s B cell lymphoma, chronic lymphocytic leukemia, Wegener’s granulomatosis, and in patients with rheumatoid arthritis who are not responding to TNF-α blockers (U.S. Food and Drug Administration, 2012). Notably, although the sole relevant trial of RTX (in the present network meta-analysis) showed that the medication had reduced the number of total gadolinium-enhancing lesions early at 12 weeks and the effects were sustained for 48 weeks, mild-to-moderate infusion-related adverse events have been repeatedly reported in several studies, particularly after the first infusion (Yamout et al., 2018, Airas et al., 2020, Hauser et al., 2008). Similarly, injection-related systemic reactions have also been reported after the first injections of OFA and OCR due to a type 2 hypersensitivity reaction, in which the release of cytokines from effector cells cause such reactions. Nevertheless, our analysis showed no difference in all safety measures between the included biological anti-CD20 agents. Future large trials based on long-term follow-up periods are needed to confirm the safety profiles of these novel medications, especially injection-related hypersensitivity events.

In the present study, the clinical efficacy and safety of OCR was supported in three trials (Hauser et al., 2017, Kappos et al., 2011). B cell depletion has been safely achieved, since the risk of serious adverse events was significantly lower compared to INF β-1a. Actually, as with other anti-CD20 therapies, B cell reconstitution and preexisting humoral immunity are relatively preserved with OCR administration because plasma cells and lymphoid stem cells lack CD20 (Coles et al., 2012). It is noteworthy that the reported results in OCR trials were based on patients with established disease, being diagnosed approximately four years earlier (the mean EDSS scores was ≥2.8). Additionally, a considerable proportion of participants were not treatment naïve. Clinically, OCR may be initiated earlier (after diagnosis) and in treatment-naïve patients to induce an early B cell depletion. This may be particularly relevant to control the dominant inflammatory reactions (over neurodegeneration) in the early stages of MS (Koudriavtseva and Mainero, 2016). Moreover, recent data suggest that switching from RTX to OCR is a safe and well-tolerated approach in RRMS patients with no significant differences in the incidence of injection-related reactions (Alvarez et al., 2019). Collectively, OCR has demonstrated promising efficacy and short-term safety outcomes in RRMS. Further long-term results may support the use of OCR in clinical practice.

The present network meta-analysis provided an early, evidence-based comparison of the efficacy and safety of anti-CD20 mAbs, considering an active MS treatment (INF β-1a) as a common comparator. Since there have been no direct comparisons of B cell therapy in RRMS, our results demonstrated no significant difference in the relative efficacy and safety of anti-CD20 mAbs compared to INF β-1a. Although several network meta-analyses of disease-modifying therapies in RRMS have been carried out (Li et al., 2020, Lucchetta et al., 2018, Tramacere et al., 2015, Fogarty et al., 2016), the present study employed strict eligibility criteria to reduce the between-study inconsistency via including consistent dosing regimens, unified follow-up periods, and clear disease classification schemes. Furthermore, we provided an updated overview of biological treatments in RRMS, including the recently published data of two large trials of OFA (Hauser et al., 2020). Finally, we focused on the comparative analysis of ant-CD20 mAbs to guide clinicians on the best performing medication in terms of two efficacy and three safety outcomes.

Despite these advantages, our study has several limitations. First, the primary efficacy outcome may have been over- or underestimated in the included RCTs. Although ARR is an important measure in RRMS, the incidence of relapses is not constant over time. In essence, the probability of relapse may be higher at the time of recruitment, but the reported relapses may be less frequent as time progresses due to the regression toward the mean phenomenon. Besides, relapses may take place on long-time intervals (years), which would go beyond the established follow-up periods of RCTs. Such limitations should be considered in future trials via employing appropriate power analyses to calculate sample sizes sufficient to detect a significant reduction in relapses (Sormani et al., 2013, Inusah et al., 2010). Second, the included trials were not powered to analyze safety, and adverse events were only reported during the periods of trials. Third, the outcomes were reported over short periods, which might further limit the reported safety results. Fifth, the authors in one trial only (Hauser et al., 2008) had explicitly provided a definition for SAEs, and no detailed assessment criteria were established for the detection of SAEs across different studies. Sixth, the small number of trials in different comparisons did not enable conducting an efficient analysis of the publication bias. Finally, the inclusion of multiple reference intervention arms has led to networks centered around multiple treatment nodes (teriflunomide, placebo, etc.).

## 5 Conclusion

In conclusion, the present network meta-analysis showed that anti-CD20 mAbs have exhibited a more favorable benefit-to-risk profile than other included agents. Based on a frequentist approach, OCR had the highest P-score ranking in terms of reducing the ARR and SAEs, with no significant differences than other anti-CD20 mAbs regarding the RR profile. Although there was no evidence of statistical heterogeneity or inconsistency and no significant disagreement was reported between direct and indirect evidence, the confidence in the estimated outcomes from the network were low to moderate for active mAbs, a matter which was primarily attributable to methodological limitations and the small number of included trials. OCR and other anti-CD20 mAbs may represent a paradigm shift in the principles of targeting B cells in RRMS; however, further long-term safety results are warranted.

## Funding

This research did not receive any specific grant from funding agencies in the public, commercial, or not-for-profit sectors.

## Compliance with ethical standards

This article does not contain any studies involving human participants performed by any of the authors.

## Declarations of interest

None.
