# Supplementary material for: The comparative efficacy and safety of anti-CD20 monoclonal antibodies for relapsing-remitting multiple sclerosis: A network meta-analysis
Source: IBRO Neurosci Rep. 2021 Aug 27;11:103–11. doi: 10.1016/j.ibneur.2021.08.003 (PMC8411244; doi:10.1016/j.ibneur.2021.08.003)
Supplement: Supplementary file 1 — Supplementary material [file mmc1.docx]

**Appendix 1**. The used search strategy in the PubMed database

#1 “rituximab” OR “ocrelizumab” OR “ofatumumab” OR “ublituximab”

#2 "trial" OR "rand*" OR "randomized" OR "randomised"

#3 "multiple sclerosis"

#4 "relapsing" OR "remitting"

#5 #1 AND #2 AND #3 AND #4

**Appendix 2**. A summary of the authors’ judgements about the risk of bias for each trial, including the following domains: the randomization process (D1), deviations from intended interventions (D2), missing outcome data (D3), measurement of the outcome (D4), and selection of the reported results (D5)


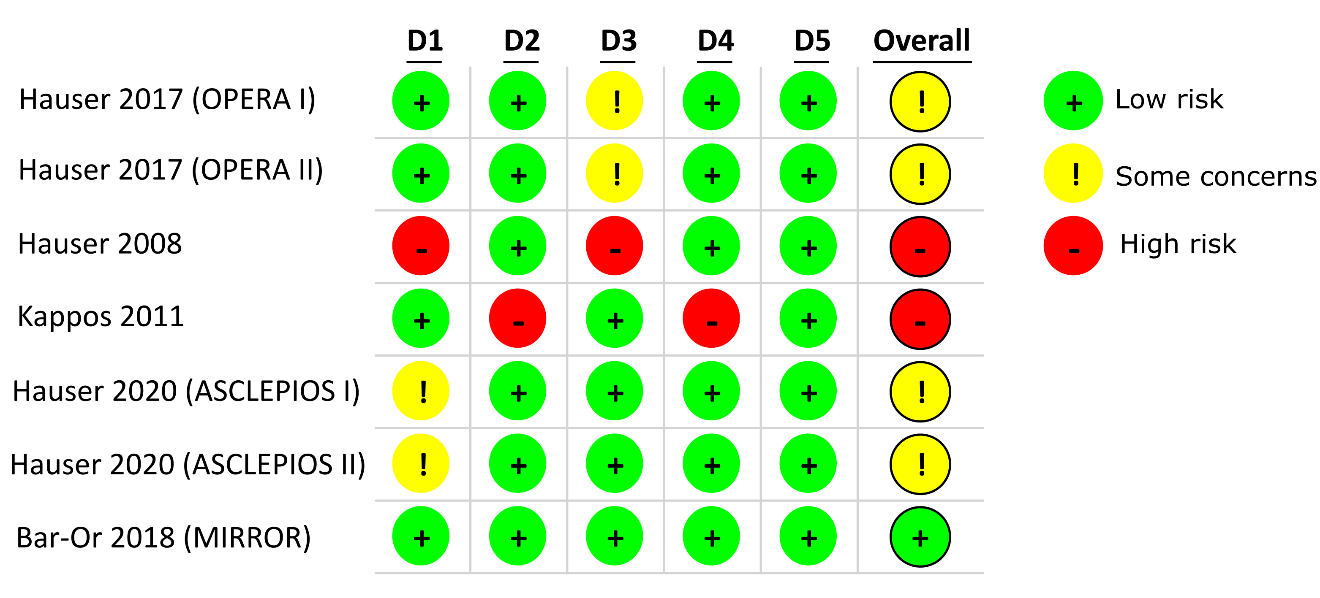


**Appendix 3**. Assessment of risk of bias

| **Unique ID** | 1 | **Study ID** | |  | | **Assessor** | M.Z.I.A and Y.A |
| --- | --- | --- | --- | --- | --- | --- | --- |
| **Ref or Label** | Hauser 2017 (OPERA I) | **Aim** | | assignment to intervention (the 'intention-to-treat' effect) | |  |  |
| **Domain** | **Signalling question** | | **Response** | | **Comments** | | |
| **Bias arising from the randomization process** | 1.1 Was the allocation sequence random? | | Y | | Randomization was performed using an interactive web-response system; therefore, the method of randomization was adequate, and the allocators had no control over the sequence generation process. | | |
|  | 1.2 Was the allocation sequence concealed until participants were enrolled and assigned to interventions? | | Y | |  |  |  |
|  | 1.3 Did baseline differences between intervention groups suggest a problem with the randomization process? | | N | | No significant differences were detected in the reported baseline characteristics. | | |
|  | **Risk of bias judgement** | | **Low** | |  | | |
| **Bias due to deviations from intended interventions** | 2.1.Were participants aware of their assigned intervention during the trial? | | N | | Patients and examining and treating investigators were unaware of the treatment assignment. | | |
|  | 2.2.Were carers and people delivering the interventions aware of participants' assigned intervention during the trial? | | N | |  |  |  |
|  | 2.3. If Y/PY/NI to 2.1 or 2.2: Were there deviations from the intended intervention that arose because of the experimental context? | | NA | |  | | |
|  | 2.4 If Y/PY to 2.3: Were these deviations likely to have affected the outcome? | | NA | |  | | |
|  | 2.5. If Y/PY/NI to 2.4: Were these deviations from intended intervention balanced between groups? | | NA | |  | | |
|  | 2.6 Was an appropriate analysis used to estimate the effect of assignment to intervention? | | Y | | The analysis of efficacy and safety outcomes was conducted in the intention-to-treat population. | | |
|  | 2.7 If N/PN/NI to 2.6: Was there potential for a substantial impact (on the result) of the failure to analyse participants in the group to which they were randomized? | | NA | |  | | |
|  | **Risk of bias judgement** | | **Low** | |  | | |
| **Bias due to missing outcome data** | 3.1 Were data for this outcome available for all, or nearly all, participants randomized? | | N | | Data was missing for 10.7% and 17.3% of patients in the OCR and IFNb1a, respectively. | | |
|  | 3.2 If N/PN/NI to 3.1: Is there evidence that result was not biased by missing outcome data? | | PY | | The authors used an intention-to-treat analysis to overcome the bias that might have emerged from patients' withdrawal. That is, sensitivity analyses of ARR were carried out for patients who had discontinued the intervention without any relapse 30 days prior to discontinuation. | | |
|  | 3.3 If N/PN to 3.2: Could missingness in the outcome depend on its true value? | | NA | |  | | |
|  | 3.4 If Y/PY/NI to 3.3: Is it likely that missingness in the outcome depended on its true value? | | NA | |  |  |  |
|  | **Risk of bias judgement** | | **Some concerns** | |  | | |
| **Bias in measurement of the outcome** | 4.1 Was the method of measuring the outcome inappropriate? | | N | |  | | |
|  | 4.2 Could measurement or ascertainment of the outcome have differed between intervention groups? | | N | |  | | |
|  | 4.3 Were outcome assessors aware of the intervention received by study participants? | | N | | Outcome assessors were unaware of the received intervention. | | |
|  | 4.4 If Y/PY/NI to 4.3: Could assessment of the outcome have been influenced by knowledge of intervention received? | | NA | |  | | |
|  | 4.5 If Y/PY/NI to 4.4: Is it likely that assessment of the outcome was influenced by knowledge of intervention received? | | NA | |  |  |  |
|  | **Risk of bias judgement** | | **Low** | |  | | |
| **Bias in selection of the reported result** | 5.1 Were the data that produced this result analysed in accordance with a pre-specified analysis plan that was finalized before unblinded outcome data were available for analysis? | | Y | | The predefined statistical analysis plan (in the study protocol) included adequate plans for handing missing outcome data, and that plan was applied in the published study. | | |
|  | 5.2 ... multiple eligible outcome measurements (e.g. scales, definitions, time points) within the outcome domain? | | N | | The reported numerical results were not selected from other multiple intervention effect estimates because the reported outcomes in the published study were similar to those reported in the study protocol (ClinicalTrials.gov Identifier: NCT01247324) | | |
|  | 5.3 ... multiple eligible analyses of the data? | | N | |  | | |
|  | **Risk of bias judgement** | | **Low** | |  | | |
| **Overall bias** | **Risk of bias judgement** | | **Some concerns** | |  | | |
|  |  |  | |  | |  |  |
|  |  |  | |  | |  |  |
| **Unique ID** | 2 | **Study ID** | |  | | **Assessor** | M.Z.I.A and Y.A |
| **Ref or Label** | Hauser 2017 (OPERA II) | **Aim** | | assignment to intervention (the 'intention-to-treat' effect) | |  |  |
| **Domain** | **Signalling question** | | **Response** | | **Comments** | | |
| **Bias arising from the randomization process** | 1.1 Was the allocation sequence random? | | Y | | Randomization was performed using an interactive web-response system; therefore, the method of randomization was adequate, and the allocators had no control over the sequence generation process. | | |
|  | 1.2 Was the allocation sequence concealed until participants were enrolled and assigned to interventions? | | Y | |  |  |  |
|  | 1.3 Did baseline differences between intervention groups suggest a problem with the randomization process? | | N | | No significant differences were detected in the reported baseline characteristics. | | |
|  | **Risk of bias judgement** | | **Low** | |  | | |
| **Bias due to deviations from intended interventions** | 2.1.Were participants aware of their assigned intervention during the trial? | | N | | Patients and examining and treating investigators were unaware of the treatment assignment. | | |
|  | 2.2.Were carers and people delivering the interventions aware of participants' assigned intervention during the trial? | | N | |  |  |  |
|  | 2.3. If Y/PY/NI to 2.1 or 2.2: Were there deviations from the intended intervention that arose because of the experimental context? | | NA | |  | | |
|  | 2.4 If Y/PY to 2.3: Were these deviations likely to have affected the outcome? | | NA | |  | | |
|  | 2.5. If Y/PY/NI to 2.4: Were these deviations from intended intervention balanced between groups? | | NA | |  | | |
|  | 2.6 Was an appropriate analysis used to estimate the effect of assignment to intervention? | | Y | | The analysis of efficacy and safety outcomes was conducted in the intention-to-treat population. | | |
|  | 2.7 If N/PN/NI to 2.6: Was there potential for a substantial impact (on the result) of the failure to analyse participants in the group to which they were randomized? | | NA | |  | | |
|  | **Risk of bias judgement** | | **Low** | |  | | |
| **Bias due to missing outcome data** | 3.1 Were data for this outcome available for all, or nearly all, participants randomized? | | N | | Data was missing for 13.7% and 23.4% of patients in the OCR and IFNb1a, respectively. | | |
|  | 3.2 If N/PN/NI to 3.1: Is there evidence that result was not biased by missing outcome data? | | PY | | The authors used an intention-to-treat analysis to overcome the bias that might have emerged from patients' withdrawal. That is, sensitivity analyses of ARR were carried out for patients who had discontinued the intervention without any relapse 30 days prior to discontinuation. | | |
|  | 3.3 If N/PN to 3.2: Could missingness in the outcome depend on its true value? | | NA | |  | | |
|  | 3.4 If Y/PY/NI to 3.3: Is it likely that missingness in the outcome depended on its true value? | | NA | |  |  |  |
|  | **Risk of bias judgement** | | **Some concerns** | |  | | |
| **Bias in measurement of the outcome** | 4.1 Was the method of measuring the outcome inappropriate? | | N | |  | | |
|  | 4.2 Could measurement or ascertainment of the outcome have differed between intervention groups? | | N | |  | | |
|  | 4.3 Were outcome assessors aware of the intervention received by study participants? | | N | | Outcome assessors were unaware of the received intervention. | | |
|  | 4.4 If Y/PY/NI to 4.3: Could assessment of the outcome have been influenced by knowledge of intervention received? | | NA | |  | | |
|  | 4.5 If Y/PY/NI to 4.4: Is it likely that assessment of the outcome was influenced by knowledge of intervention received? | | NA | |  |  |  |
|  | **Risk of bias judgement** | | **Low** | |  | | |
| **Bias in selection of the reported result** | 5.1 Were the data that produced this result analysed in accordance with a pre-specified analysis plan that was finalized before unblinded outcome data were available for analysis? | | Y | | The predefined statistical analysis plan (in the study protocol) included adequate plans for handing missing outcome data, and that plan was applied in the published study. | | |
|  | 5.2 ... multiple eligible outcome measurements (e.g. scales, definitions, time points) within the outcome domain? | | N | | The reported numerical results were not selected from other multiple intervention effect estimates because the reported outcomes in the published study were similar to those reported in the study protocol (ClinicalTrials.gov Identifier: NCT01247324). | | |
|  | 5.3 ... multiple eligible analyses of the data? | | N | |  | | |
|  | **Risk of bias judgement** | | **Low** | |  | | |
| **Overall bias** | **Risk of bias judgement** | | **Some concerns** | |  | | |
|  |  |  | |  | |  |  |
|  |  |  | |  | |  |  |
| **Unique ID** | 3 | **Study ID** | |  | | **Assessor** | M.Z.I.A and Y.A |
| **Ref or Label** | Hauser 2008 | **Aim** | | assignment to intervention (the 'intention-to-treat' effect) | |  |  |
| **Domain** | **Signalling question** | | **Response** | | **Comments** | | |
| **Bias arising from the randomization process** | 1.1 Was the allocation sequence random? | | PY | | The authors reported that the study was randomized study; however, they did not mention the method of randomization. Furthermore, the authors did not report information about allocation concealment. | | |
|  | 1.2 Was the allocation sequence concealed until participants were enrolled and assigned to interventions? | | NI | |  |  |  |
|  | 1.3 Did baseline differences between intervention groups suggest a problem with the randomization process? | | PY | | Although baseline characteristics were balanced, the percentage of patients with gadolinium-enhancing lesions was significantly lower in the RTX group than in the placebo group. This might indicate problems in the randomization process, which might have led to bias in the intervention effect estimate. | | |
|  | **Risk of bias judgement** | | **High** | |  | | |
| **Bias due to deviations from intended interventions** | 2.1.Were participants aware of their assigned intervention during the trial? | | N | | Each site of the trial setting had both an examining investigator (the efficacy assessor) and a treating investigator (the safety assessor) | | |
|  | 2.2.Were carers and people delivering the interventions aware of participants' assigned intervention during the trial? | | N | |  |  |  |
|  | 2.3. If Y/PY/NI to 2.1 or 2.2: Were there deviations from the intended intervention that arose because of the experimental context? | | NA | |  | | |
|  | 2.4 If Y/PY to 2.3: Were these deviations likely to have affected the outcome? | | NA | |  | | |
|  | 2.5. If Y/PY/NI to 2.4: Were these deviations from intended intervention balanced between groups? | | NA | |  | | |
|  | 2.6 Was an appropriate analysis used to estimate the effect of assignment to intervention? | | Y | |  | | |
|  | 2.7 If N/PN/NI to 2.6: Was there potential for a substantial impact (on the result) of the failure to analyse participants in the group to which they were randomized? | | NA | |  | | |
|  | **Risk of bias judgement** | | **Low** | |  | | |
| **Bias due to missing outcome data** | 3.1 Were data for this outcome available for all, or nearly all, participants randomized? | | N | | The rates of dropouts over 48 weeks were relatively high in the active treatment arm (23.9%) and very high in the placebo arm (40%). | | |
|  | 3.2 If N/PN/NI to 3.1: Is there evidence that result was not biased by missing outcome data? | | PN | | Data analysis was based on an intention-to-treat basis (using the Last observation carried forward [LOCF]); however, the baseline differences between study groups as well as the differences in data missingness might have resulted in a bias due to missing outcome data. | | |
|  | 3.3 If N/PN to 3.2: Could missingness in the outcome depend on its true value? | | Y | | There was a significant difference between study groups in proportions of missing outcome data. | | |
|  | 3.4 If Y/PY/NI to 3.3: Is it likely that missingness in the outcome depended on its true value? | | Y | |  |  |  |
|  | **Risk of bias judgement** | | **High** | |  | | |
| **Bias in measurement of the outcome** | 4.1 Was the method of measuring the outcome inappropriate? | | N | |  | | |
|  | 4.2 Could measurement or ascertainment of the outcome have differed between intervention groups? | | N | |  | | |
|  | 4.3 Were outcome assessors aware of the intervention received by study participants? | | N | |  | | |
|  | 4.4 If Y/PY/NI to 4.3: Could assessment of the outcome have been influenced by knowledge of intervention received? | | NA | |  | | |
|  | 4.5 If Y/PY/NI to 4.4: Is it likely that assessment of the outcome was influenced by knowledge of intervention received? | | NA | |  |  |  |
|  | **Risk of bias judgement** | | **Low** | |  | | |
| **Bias in selection of the reported result** | 5.1 Were the data that produced this result analysed in accordance with a pre-specified analysis plan that was finalized before unblinded outcome data were available for analysis? | | Y | | The predefined statistical analysis plan (in the study protocol) included adequate plans for handing missing outcome data, and that plan was applied in the published study. | | |
|  | 5.2 ... multiple eligible outcome measurements (e.g. scales, definitions, time points) within the outcome domain? | | N | |  | | |
|  | 5.3 ... multiple eligible analyses of the data? | | N | |  | | |
|  | **Risk of bias judgement** | | **Low** | |  | | |
| **Overall bias** | **Risk of bias judgement** | | **High** | |  | | |
|  |  |  | |  | |  |  |
|  |  |  | |  | |  |  |
| **Unique ID** | 4 | **Study ID** | |  | | **Assessor** | M.Z.I.A and Y.A |
| **Ref or Label** | Kappos 2011 | **Aim** | | assignment to intervention (the 'intention-to-treat' effect) | |  |  |
| **Domain** | **Signalling question** | | **Response** | | **Comments** | | |
| **Bias arising from the randomization process** | 1.1 Was the allocation sequence random? | | Y | |  | | |
|  | 1.2 Was the allocation sequence concealed until participants were enrolled and assigned to interventions? | | Y | |  |  |  |
|  | 1.3 Did baseline differences between intervention groups suggest a problem with the randomization process? | | N | |  | | |
|  | **Risk of bias judgement** | | **Low** | |  | | |
| **Bias due to deviations from intended interventions** | 2.1.Were participants aware of their assigned intervention during the trial? | | N | | Treatment assignment was masked for patients during the trial. However, in the IFNba1 group, raters were only blinded to allocation, but not other subsequent procedures. Therefore, comparisons of OCR and IFNb1a groups were deemed exploratory. | | |
|  | 2.2.Were carers and people delivering the interventions aware of participants' assigned intervention during the trial? | | PY | |  |  |  |
|  | 2.3. If Y/PY/NI to 2.1 or 2.2: Were there deviations from the intended intervention that arose because of the experimental context? | | N | |  | | |
|  | 2.4 If Y/PY to 2.3: Were these deviations likely to have affected the outcome? | | NA | |  | | |
|  | 2.5. If Y/PY/NI to 2.4: Were these deviations from intended intervention balanced between groups? | | NA | |  | | |
|  | 2.6 Was an appropriate analysis used to estimate the effect of assignment to intervention? | | PN | | An intention-to-treat (ITT) analysis was not performed on the ARR outcome; yet, it was applied on relapse-free events and on safety outcomes. | | |
|  | 2.7 If N/PN/NI to 2.6: Was there potential for a substantial impact (on the result) of the failure to analyse participants in the group to which they were randomized? | | PY | |  | | |
|  | **Risk of bias judgement** | | **High** | |  | | |
| **Bias due to missing outcome data** | 3.1 Were data for this outcome available for all, or nearly all, participants randomized? | | Y | | The proportion of patients with no available data at the study end point were 10.9% in the IFN1a group, 12.5% in the OCR group, and 11.1% in the placebo group. | | |
|  | 3.2 If N/PN/NI to 3.1: Is there evidence that result was not biased by missing outcome data? | | NA | |  | | |
|  | 3.3 If N/PN to 3.2: Could missingness in the outcome depend on its true value? | | NA | |  | | |
|  | 3.4 If Y/PY/NI to 3.3: Is it likely that missingness in the outcome depended on its true value? | | NA | |  |  |  |
|  | **Risk of bias judgement** | | **Low** | |  | | |
| **Bias in measurement of the outcome** | 4.1 Was the method of measuring the outcome inappropriate? | | N | |  | | |
|  | 4.2 Could measurement or ascertainment of the outcome have differed between intervention groups? | | N | |  | | |
|  | 4.3 Were outcome assessors aware of the intervention received by study participants? | | Y | | Only the raters in the interferon beta-1a group were blinded to allocation; therefore, outcome assessors in this particular group were aware of the intervention received. | | |
|  | 4.4 If Y/PY/NI to 4.3: Could assessment of the outcome have been influenced by knowledge of intervention received? | | Y | | Outcome assessment might have been affected by knowledge of the provided intervention. | | |
|  | 4.5 If Y/PY/NI to 4.4: Is it likely that assessment of the outcome was influenced by knowledge of intervention received? | | Y | |  |  |  |
|  | **Risk of bias judgement** | | **High** | |  | | |
| **Bias in selection of the reported result** | 5.1 Were the data that produced this result analysed in accordance with a pre-specified analysis plan that was finalized before unblinded outcome data were available for analysis? | | Y | |  | | |
|  | 5.2 ... multiple eligible outcome measurements (e.g. scales, definitions, time points) within the outcome domain? | | N | |  | | |
|  | 5.3 ... multiple eligible analyses of the data? | | N | |  | | |
|  | **Risk of bias judgement** | | **Low** | |  | | |
| **Overall bias** | **Risk of bias judgement** | | **High** | |  | | |
|  |  |  | |  | |  |  |
|  |  |  | |  | |  |  |
| **Unique ID** | 5 | **Study ID** | |  | | **Assessor** | M.Z.I.A and Y.A |
| **Ref or Label** | Hauser 2020 (ASCLEPIOS I) | **Aim** | | assignment to intervention (the 'intention-to-treat' effect) | |  |  |
| **Domain** | **Signalling question** | | **Response** | | **Comments** | | |
| **Bias arising from the randomization process** | 1.1 Was the allocation sequence random? | | PY | | The authors reported that the study was randomized study; however, they did not mention the method of randomization. Furthermore, the authors did not report information about allocation concealment. | | |
|  | 1.2 Was the allocation sequence concealed until participants were enrolled and assigned to interventions? | | NI | |  |  |  |
|  | 1.3 Did baseline differences between intervention groups suggest a problem with the randomization process? | | N | |  | | |
|  | **Risk of bias judgement** | | **Some concerns** | |  | | |
| **Bias due to deviations from intended interventions** | 2.1.Were participants aware of their assigned intervention during the trial? | | N | | The investigators, the sponsors, the statisticians, and the steering committee were all unaware of treatment assignment throughout the trial. | | |
|  | 2.2.Were carers and people delivering the interventions aware of participants' assigned intervention during the trial? | | N | |  |  |  |
|  | 2.3. If Y/PY/NI to 2.1 or 2.2: Were there deviations from the intended intervention that arose because of the experimental context? | | NA | |  | | |
|  | 2.4 If Y/PY to 2.3: Were these deviations likely to have affected the outcome? | | NA | |  | | |
|  | 2.5. If Y/PY/NI to 2.4: Were these deviations from intended intervention balanced between groups? | | NA | |  | | |
|  | 2.6 Was an appropriate analysis used to estimate the effect of assignment to intervention? | | Y | | Efficacy analyses were conducted based on the intention-to-treat principle | | |
|  | 2.7 If N/PN/NI to 2.6: Was there potential for a substantial impact (on the result) of the failure to analyse participants in the group to which they were randomized? | | NA | |  | | |
|  | **Risk of bias judgement** | | **Low** | |  | | |
| **Bias due to missing outcome data** | 3.1 Were data for this outcome available for all, or nearly all, participants randomized? | | PY | | No data was available for 10.5% of patients in the OFA group and 18.6% in the teriflunomide group. | | |
|  | 3.2 If N/PN/NI to 3.1: Is there evidence that result was not biased by missing outcome data? | | NA | |  | | |
|  | 3.3 If N/PN to 3.2: Could missingness in the outcome depend on its true value? | | NA | |  | | |
|  | 3.4 If Y/PY/NI to 3.3: Is it likely that missingness in the outcome depended on its true value? | | NA | |  |  |  |
|  | **Risk of bias judgement** | | **Low** | |  | | |
| **Bias in measurement of the outcome** | 4.1 Was the method of measuring the outcome inappropriate? | | N | |  | | |
|  | 4.2 Could measurement or ascertainment of the outcome have differed between intervention groups? | | N | |  | | |
|  | 4.3 Were outcome assessors aware of the intervention received by study participants? | | N | |  | | |
|  | 4.4 If Y/PY/NI to 4.3: Could assessment of the outcome have been influenced by knowledge of intervention received? | | NA | |  | | |
|  | 4.5 If Y/PY/NI to 4.4: Is it likely that assessment of the outcome was influenced by knowledge of intervention received? | | NA | |  |  |  |
|  | **Risk of bias judgement** | | **Low** | |  | | |
| **Bias in selection of the reported result** | 5.1 Were the data that produced this result analysed in accordance with a pre-specified analysis plan that was finalized before unblinded outcome data were available for analysis? | | Y | | The predefined statistical analysis plan (in the study protocol) included adequate plans for handing missing outcome data, and that plan was applied in the published study. | | |
|  | 5.2 ... multiple eligible outcome measurements (e.g. scales, definitions, time points) within the outcome domain? | | N | | The reported numerical results were not selected from other multiple intervention effect estimates because the reported outcomes in the published study were similar to those reported in the study protocol (ClinicalTrials.gov Identifier: NCT02792218) | | |
|  | 5.3 ... multiple eligible analyses of the data? | | N | |  | | |
|  | **Risk of bias judgement** | | **Low** | |  | | |
| **Overall bias** | **Risk of bias judgement** | | **Some concerns** | |  | | |
|  |  |  | |  | |  |  |
|  |  |  | |  | |  |  |
| **Unique ID** | 6 | **Study ID** | |  | | **Assessor** | M.Z.I.A and Y.A |
| **Ref or Label** | Hauser 2020 (ASCLEPIOS II) | **Aim** | | assignment to intervention (the 'intention-to-treat' effect) | |  |  |
| **Domain** | **Signalling question** | | **Response** | | **Comments** | | |
| **Bias arising from the randomization process** | 1.1 Was the allocation sequence random? | | PY | | The authors reported that the study was randomized study; however, they did not mention the method of randomization. Furthermore, the authors did not report information about allocation concealment. | | |
|  | 1.2 Was the allocation sequence concealed until participants were enrolled and assigned to interventions? | | NI | |  |  |  |
|  | 1.3 Did baseline differences between intervention groups suggest a problem with the randomization process? | | N | |  | | |
|  | **Risk of bias judgement** | | **Some concerns** | |  | | |
| **Bias due to deviations from intended interventions** | 2.1.Were participants aware of their assigned intervention during the trial? | | N | | The investigators, the sponsors, the statisticians, and the steering committee were all unaware of treatment assignment throughout the trial. | | |
|  | 2.2.Were carers and people delivering the interventions aware of participants' assigned intervention during the trial? | | N | |  |  |  |
|  | 2.3. If Y/PY/NI to 2.1 or 2.2: Were there deviations from the intended intervention that arose because of the experimental context? | | NA | |  | | |
|  | 2.4 If Y/PY to 2.3: Were these deviations likely to have affected the outcome? | | NA | |  | | |
|  | 2.5. If Y/PY/NI to 2.4: Were these deviations from intended intervention balanced between groups? | | NA | |  | | |
|  | 2.6 Was an appropriate analysis used to estimate the effect of assignment to intervention? | | Y | | Efficacy analyses were conducted based on the intention-to-treat principle | | |
|  | 2.7 If N/PN/NI to 2.6: Was there potential for a substantial impact (on the result) of the failure to analyse participants in the group to which they were randomized? | | NA | |  | | |
|  | **Risk of bias judgement** | | **Low** | |  | | |
| **Bias due to missing outcome data** | 3.1 Were data for this outcome available for all, or nearly all, participants randomized? | | PY | | No data was available for 17.5% of patients in the OFA group and 17.9% in the teriflunomide group. | | |
|  | 3.2 If N/PN/NI to 3.1: Is there evidence that result was not biased by missing outcome data? | | NA | |  | | |
|  | 3.3 If N/PN to 3.2: Could missingness in the outcome depend on its true value? | | NA | |  | | |
|  | 3.4 If Y/PY/NI to 3.3: Is it likely that missingness in the outcome depended on its true value? | | NA | |  |  |  |
|  | **Risk of bias judgement** | | **Low** | |  | | |
| **Bias in measurement of the outcome** | 4.1 Was the method of measuring the outcome inappropriate? | | N | |  | | |
|  | 4.2 Could measurement or ascertainment of the outcome have differed between intervention groups? | | N | |  | | |
|  | 4.3 Were outcome assessors aware of the intervention received by study participants? | | N | |  | | |
|  | 4.4 If Y/PY/NI to 4.3: Could assessment of the outcome have been influenced by knowledge of intervention received? | | NA | |  | | |
|  | 4.5 If Y/PY/NI to 4.4: Is it likely that assessment of the outcome was influenced by knowledge of intervention received? | | NA | |  |  |  |
|  | **Risk of bias judgement** | | **Low** | |  | | |
| **Bias in selection of the reported result** | 5.1 Were the data that produced this result analysed in accordance with a pre-specified analysis plan that was finalized before unblinded outcome data were available for analysis? | | Y | | The predefined statistical analysis plan (in the study protocol) included adequate plans for handing missing outcome data, and that plan was applied in the published study. | | |
|  | 5.2 ... multiple eligible outcome measurements (e.g. scales, definitions, time points) within the outcome domain? | | N | | The reported numerical results were not selected from other multiple intervention effect estimates because the reported outcomes in the published study were similar to those reported in the study protocol (ClinicalTrials.gov Identifier: NCT02792231) | | |
|  | 5.3 ... multiple eligible analyses of the data? | | N | |  | | |
|  | **Risk of bias judgement** | | **Low** | |  | | |
| **Overall bias** | **Risk of bias judgement** | | **Some concerns** | |  | | |
|  |  |  | |  | |  |  |
|  |  |  | |  | |  |  |
| **Unique ID** | 7 | **Study ID** | |  | | **Assessor** | M.Z.I.A and Y.A |
| **Ref or Label** | Bar-Or 2018 (MIRROR) | **Aim** | | assignment to intervention (the 'intention-to-treat' effect) | |  |  |
| **Domain** | **Signalling question** | | **Response** | | **Comments** | | |
| **Bias arising from the randomization process** | 1.1 Was the allocation sequence random? | | Y | | Randomization was computer generated; therefore, the method of randomization was adequate, and the allocators had no control over the sequence generation process. Patients remained blinded of the intervention throughout the study. | | |
|  | 1.2 Was the allocation sequence concealed until participants were enrolled and assigned to interventions? | | Y | |  |  |  |
|  | 1.3 Did baseline differences between intervention groups suggest a problem with the randomization process? | | N | |  | | |
|  | **Risk of bias judgement** | | **Low** | |  | | |
| **Bias due to deviations from intended interventions** | 2.1.Were participants aware of their assigned intervention during the trial? | | N | |  | | |
|  | 2.2.Were carers and people delivering the interventions aware of participants' assigned intervention during the trial? | | N | |  |  |  |
|  | 2.3. If Y/PY/NI to 2.1 or 2.2: Were there deviations from the intended intervention that arose because of the experimental context? | | NA | |  | | |
|  | 2.4 If Y/PY to 2.3: Were these deviations likely to have affected the outcome? | | NA | |  | | |
|  | 2.5. If Y/PY/NI to 2.4: Were these deviations from intended intervention balanced between groups? | | NA | |  | | |
|  | 2.6 Was an appropriate analysis used to estimate the effect of assignment to intervention? | | Y | |  | | |
|  | 2.7 If N/PN/NI to 2.6: Was there potential for a substantial impact (on the result) of the failure to analyse participants in the group to which they were randomized? | | NA | |  | | |
|  | **Risk of bias judgement** | | **Low** | |  | | |
| **Bias due to missing outcome data** | 3.1 Were data for this outcome available for all, or nearly all, participants randomized? | | Y | |  | | |
|  | 3.2 If N/PN/NI to 3.1: Is there evidence that result was not biased by missing outcome data? | | NA | |  | | |
|  | 3.3 If N/PN to 3.2: Could missingness in the outcome depend on its true value? | | NA | |  | | |
|  | 3.4 If Y/PY/NI to 3.3: Is it likely that missingness in the outcome depended on its true value? | | NA | |  |  |  |
|  | **Risk of bias judgement** | | **Low** | |  | | |
| **Bias in measurement of the outcome** | 4.1 Was the method of measuring the outcome inappropriate? | | N | |  | | |
|  | 4.2 Could measurement or ascertainment of the outcome have differed between intervention groups? | | N | |  | | |
|  | 4.3 Were outcome assessors aware of the intervention received by study participants? | | N | |  | | |
|  | 4.4 If Y/PY/NI to 4.3: Could assessment of the outcome have been influenced by knowledge of intervention received? | | NA | |  | | |
|  | 4.5 If Y/PY/NI to 4.4: Is it likely that assessment of the outcome was influenced by knowledge of intervention received? | | NA | |  |  |  |
|  | **Risk of bias judgement** | | **Low** | |  | | |
| **Bias in selection of the reported result** | 5.1 Were the data that produced this result analysed in accordance with a pre-specified analysis plan that was finalized before unblinded outcome data were available for analysis? | | Y | | The predefined statistical analysis plan (in the study protocol) included adequate plans for handing missing outcome data, and that plan was applied in the published study. | | |
|  | 5.2 ... multiple eligible outcome measurements (e.g. scales, definitions, time points) within the outcome domain? | | N | | The reported numerical results were not selected from other multiple intervention effect estimates because the reported outcomes in the published study were similar to those reported in the study protocol (ClinicalTrials.gov Identifier: NCT01457924) | | |
|  | 5.3 ... multiple eligible analyses of the data? | | N | |  | | |
|  | **Risk of bias judgement** | | **Low** | |  | | |
| **Overall bias** | **Risk of bias judgement** | | **Low** | |  | | |

**Appendix 4: Summary of Findings table summarizing the relative effects of the network meta-analysis, the absolute effects of interventions and the comparator arm, and the results of the certainty of evidence.**

| **Outcomes/interventions** | **Relative effect* (95%CI)** | **Anticipated absolute effect** (95%CI)** | | | **Certainty of evidence (GRADE)***** |
| --- | --- | --- | --- | --- | --- |
|  |  | **Without intervention** | **With intervention** | **Difference** |  |
| **Annualized relapse rate** | | | | | |
| Ocrelizumab  (3 RCTs; 3421 participants) | **RR 0.56**  (0.50; 0.64) | 291 per 1000 | 164 per 1000 | 128 more per 1000  (100 more to 155 more) | ⨁⨁◯◯  **Low**  Due to study design limitations^1^ |
| Ofatumumab  (No direct evidence; Indirect evidence only) | **RR 0.75**  (0.32; 1.77) | 291 per 1000 | 106 per 1000 | 185 more per 1000  (160 more to 211 more) | ⨁⨁⨁⨁  **High** |
| Rituximab  (No direct evidence; Indirect evidence only) | **RR 0.88**  (0.54; 1.45) | 291 per 1000 | 406 per 1000 | 114 fewer per 1000  (234 fewer to 4 fewer) | ⨁⨁◯◯  **Low**  Due to study design limitations^2^, and imprecision^3^ |
| Teriflunomide  (No direct evidence; Indirect evidence only) | **RR 1.56**  (1.11; 2.19) | 291 per 1000 | 235 per 1000 | 56 more per 1000  (27 more to 85 more) | ⨁⨁⨁◯  **Moderate**  Due to indirectness^4^ |
| Placebo  (1 RCT; 108 participants) | **RR 1.69**  (0.71; 4.01) | 291 per 1000 | 551 per 1000 | 260 fewer per 1000  (339 fewer to 178 fewer) | ⨁◯◯◯  **Very low**  Due to study design limitations^5^ and imprecision^3^ |
| IFNb1a  (Reference comparator) | Reference comparator | Non-estimable | Non-estimable | Non-estimable | Reference comparator |
| **Serious AE** | | | | | |
| Ocrelizumab  (3 RCTs; 3421 participants) | **RR 0.11**  (0.00; 2.90) | 84 per 1000 | 14 per 1000 | 70 more per 1000  (51 more to 91 more) | ⨁⨁◯◯  **Low**  Due to study design limitations^1^ |
| Ofatumumab  (No direct evidence; Indirect evidence only) | **RR 0.26**  (0.03; 2.05) | 84 per 1000 | 88 per 1000 | 4 fewer per 1000  (30 fewer to 22 more) | ⨁⨁⨁⨁  **High** |
| Rituximab  (No direct evidence; Indirect evidence only) | **RR 0.24**  (0.04; 1.44) | 84 per 1000 | 130 per 1000 | 46 fewer per 1000  (147 fewer to 17 more) | ⨁⨁◯◯  **Low**  Due to study design limitations^2^, and imprecision^3^ |
| Teriflunomide  (No direct evidence; Indirect evidence only) | **RR 0.13**  (0.00; 3.38) | 84 per 1000 | 79 per 1000 | 5 more per 1000  (20 fewer to 31 more) | ⨁⨁⨁◯  **Moderate**  Due to indirectness^4^ |
| Placebo  (1 RCT; 108 participants) | **RR 0.17**  (0.09; 0.30) | 84 per 1000 | 45 per 1000 | 39 more per 1000  (10 fewer to 69 more) | ⨁◯◯◯  **Very low**  Due to study design limitations^5^ and imprecision^3^ |
| IFNb1a  (Reference comparator) | Reference comparator | Non-estimable | Non-estimable | Non-estimable | Reference comparator |
| **Relapse-free events** | | | | | |
| Ocrelizumab  (3 RCTs; 3421 participants) | **OR 2.47**  (2.00; 3.05) | 309 per 1000 | 509 per 1000 | 200 fewer per 1000  (246 fewer to 153 fewer) | ⨁⨁◯◯  **Low**  Due to study design limitations^1^ |
| Ofatumumab  (No direct evidence; Indirect evidence only) | **OR 0.79**  (0.20; 3.19) | 309 per 1000 | 801 per 1000 | 492 fewer per 1000  (531 fewer to 450 fewer) | ⨁⨁⨁⨁  **High** |
| Rituximab  (No direct evidence; Indirect evidence only) | **OR 1.33**  (0.37; 4.82) | 309 per 1000 | 855 per 1000 | 546 fewer per 1000  (618 fewer to 440 fewer) | ⨁⨁◯◯  **Low**  Due to study design limitations^2^, and imprecision^3^ |
| Teriflunomide  (No direct evidence; Indirect evidence only) | **OR 0.43**  (0.19; 1.01) | 309 per 1000 | 593 per 1000 | 284 fewer per 1000  (328 fewer to 238 fewer) | ⨁⨁⨁◯  **Moderate**  Due to indirectness^4^ |
| Placebo  (1 RCT; 108 participants) | **OR 0.29**  (0.07; 1.18) | 309 per 1000 | 712 per 1000 | 403 fewer per 1000  (475 fewer to 321 fewer) | ⨁⨁◯◯  **Low**  Due to study design limitations^5^ |
| IFNb1a  (Reference comparator) | Reference comparator | Non-estimable | Non-estimable | Non-estimable | Reference comparator |
| **Discontinuation due to adverse events** | | | | | |
| Ocrelizumab  (3 RCTs; 3421 participants) | **RR 0.60**  (0.39; 0.93) | 59 per 1000 | 35 per 1000 | 24 more per 1000  (4 more to 44 more) | ⨁⨁◯◯  **Low**  Due to study design limitations^1^ |
| Ofatumumab  (No direct evidence; Indirect evidence only) | **RR 0.38**  (0.01; 21.26) | 59 per 1000 | 55 per 1000 | 4 more per 1000  (17 fewer to 25 more) | ⨁⨁⨁⨁  **High** |
| Rituximab  (No direct evidence; Indirect evidence only) | **RR 0.13**  (0.00; 4.09) | 59 per 1000 | 43 per 1000 | 16 more per 1000  (62 fewer to 49 more) | ⨁⨁◯◯  **Low**  Due to study design limitations^2^, and imprecision^3^ |
| Teriflunomide  (No direct evidence; Indirect evidence only) | **RR 0.18**  (0.01; 3.34) | 59 per 1000 | 52 per 1000 | 7 more per 1000  (14 fewer to 28 more) | ⨁⨁⨁◯  **Moderate**  Due to indirectness^4^ |
| Placebo  (1 RCT; 108 participants) | **RR 0.35**  (0.01; 19.84) | 59 per 1000 | 13 per 1000 | 46 more per 1000  (10 more to 66 more) | ⨁◯◯◯  **Very low**  Due to study design limitations^5^ and imprecision^3^ |
| IFNb1a  (Reference comparator) | Reference comparator | Non-estimable | Non-estimable | Non-estimable | Reference comparator |
| **Any adverse event** | | | | | |
| Ocrelizumab  (3 RCTs; 3421 participants) | **RR 1.00**  (0.96; 1.05) | 816 per 1000 | 819 per 1000 | 3 fewer per 1000  (40 fewer to 33 more) | ⨁⨁◯◯  **Low**  Due to study design limitations^1^ |
| Ofatumumab  (No direct evidence; Indirect evidence only) | **RR 0.97**  (0.61; 1.55) | 816 per 1000 | 826 per 1000 | 10 fewer per 1000  (45 fewer to 25 more) | ⨁⨁⨁⨁  **High** |
| Rituximab  (No direct evidence; Indirect evidence only) | **RR 1.18**  (0.93; 1.51) | 816 per 1000 | 986 per 1000 | 170 fewer per 1000  (199 fewer to 102 fewer) | ⨁⨁⨁◯  **Moderate**  Due to study design limitations^2^ |
| Teriflunomide  (No direct evidence; Indirect evidence only) | **RR 1.19**  (0.94; 1.51) | 816 per 1000 | 842 per 1000 | 26 fewer per 1000  (61 fewer to 9 more) | ⨁⨁⨁◯  **Moderate**  Due to indirectness^4^ |
| Placebo  (1 RCT; 108 participants) | **RR 0.98**  (0.61; 1.57) | 816 per 1000 | 734 per 1000 | 82 more per 1000  (13 more to 161 more) | ⨁⨁◯◯  **Low**  Due to study design limitations^5^ |
| IFNb1a  (Reference comparator) | Reference comparator | Non-estimable | Non-estimable | Non-estimable | Reference comparator |

**Table definitions**

* Estimates of the network meta-analysis are reported as risk ratios (RRs) and their respective 95% confidence intervals (95%CI) for annualized relapse rates (ARRs) and safety outcomes, whereas relapse-free events were expressed as odds ratio (OR) and 95%CI. The outcomes are presented based on a frequentiest network meta-analysis approach.

** Anticipated absolute effects indicates the absolute risks in the intervention groups and the reference comparator (IFNb1a), as well as the difference in absolute risks between different comparisons.

*** High quality indicates that we are very confident that the true effect is almost similar to the estimated effect; Moderate quality indicates that we are moderately confident that the true effect is likely close to the estimated one; Low quality indicates that the confidence in the estimated effect is limited, and the true effect is likely to be substantially different from the true effect; Very low quality indicates that we have very little confidence in the estimated effect.

**Explanatory footnotes**

1 Downgraded two levels due to limitations in the study design: crucial limitations for two criteria (deviations from intended interventions & measurement of the outcome) and a potential limitation that is likely to impact the estimated effect (missing outcome data).

2 Downgraded one level due to limitations in the study design: randomization process & missing outcome data

3 Downgraded one level: Due to wide confidence interval

4 Downgraded one level due to serious indirectness: the indirect evidence for the comparison emerges from a second order loop via Ofatumumab and placebo

5 Downgraded two levels due to limitations in the study design: deviations from intended interventions & measurement of the outcome.

**Appendix 5**: The results heterogeneity and inconsistency assessment.

| **Variable** | **Total heterogeneity** | | | **Within-design heterogeneity** | | | | **Between-designs inconsistency** | | |
| --- | --- | --- | --- | --- | --- | --- | --- | --- | --- | --- |
|  | **Q** | **D.F** | **p** | ***I^2^* (%)** | **Q** | **D.F** | **p** | **Q** | **D.F** | **p** |
| ARR | 3.87 | 3 | 0.276 | 22.4 | 1.89 | 2 | 0.389 | 1.98 | 1 | 0.160 |
| Serious adverse events | 1.25 | 3 | 0.740 | 0 | 0.38 | 2 | 0.825 | 0.87 | 1 | 0.351 |
| Relapse-free events | 1.23 | 3 | 0.747 | 0 | 0.98 | 2 | 0.611 | 0.24 | 1 | 0.622 |
| Any adverse event | 0.66 | 3 | 0.882 | 0 | 0.24 | 2 | 0.887 | 0.42 | 1 | 0.517 |
| Discontinuation due to adverse events | 1.33 | 3 | 0.722 | 0 | 0.3 | 2 | 0.859 | 1.03 | 1 | 0.311 |

**Appendix 6:** Assessment of the transitivity assumption across studies

**6.1. Age**

**
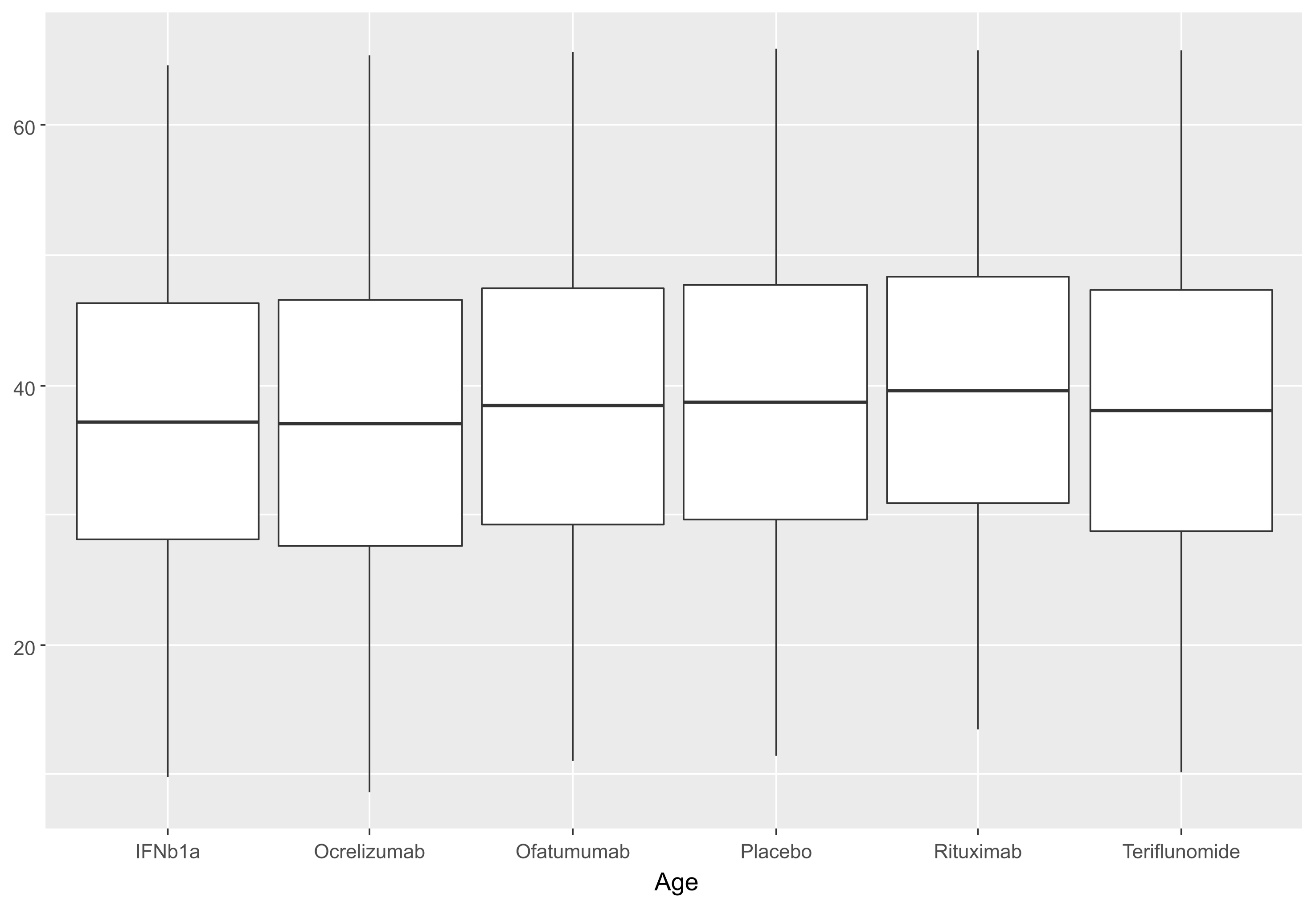
**

**6.2. Time since symptom onset**

**
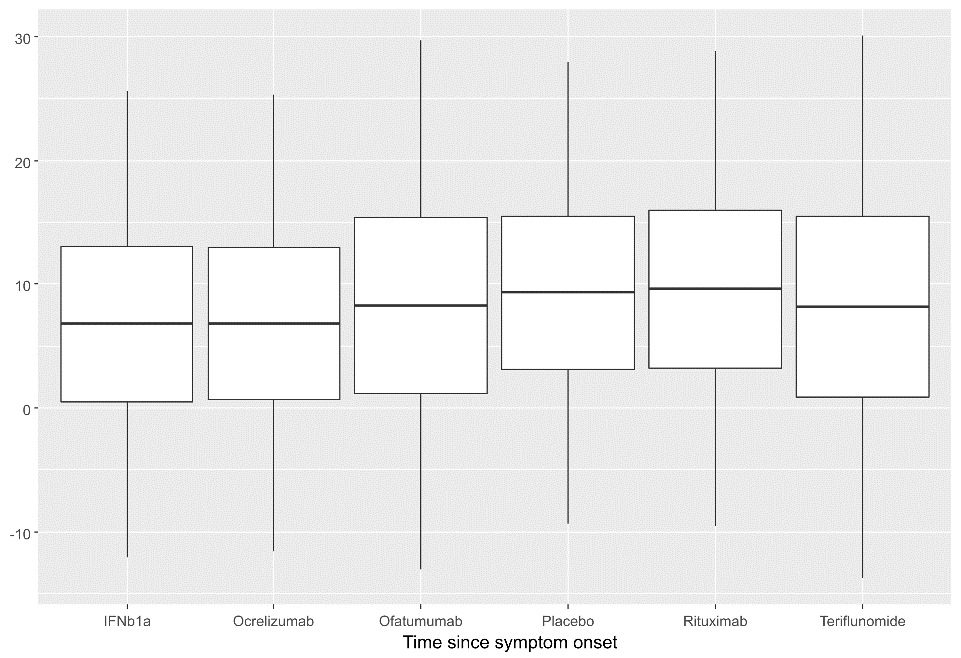
**

**6.3. Time since diagnosis**

**
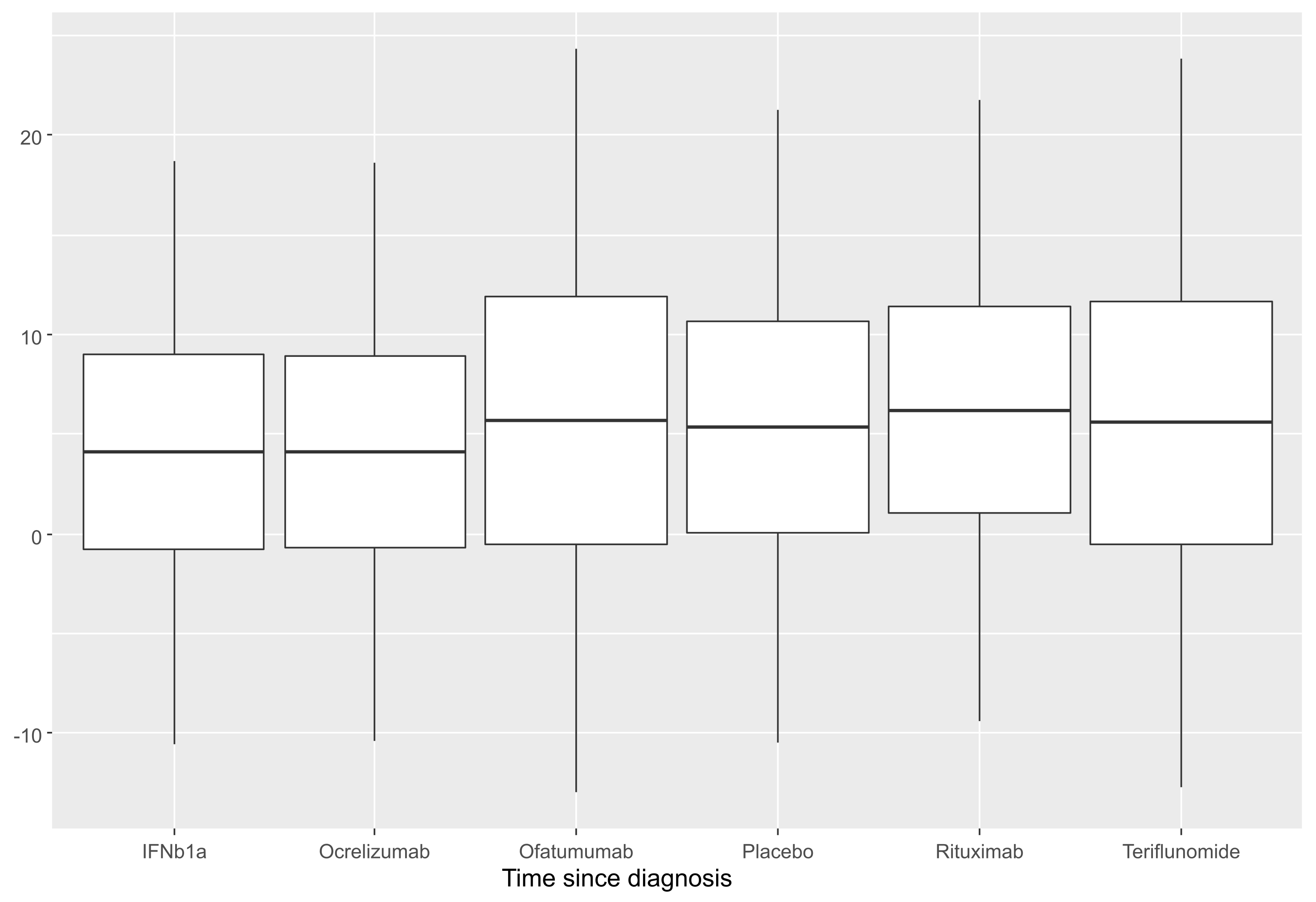
**

**6.4. EDSS score**

**
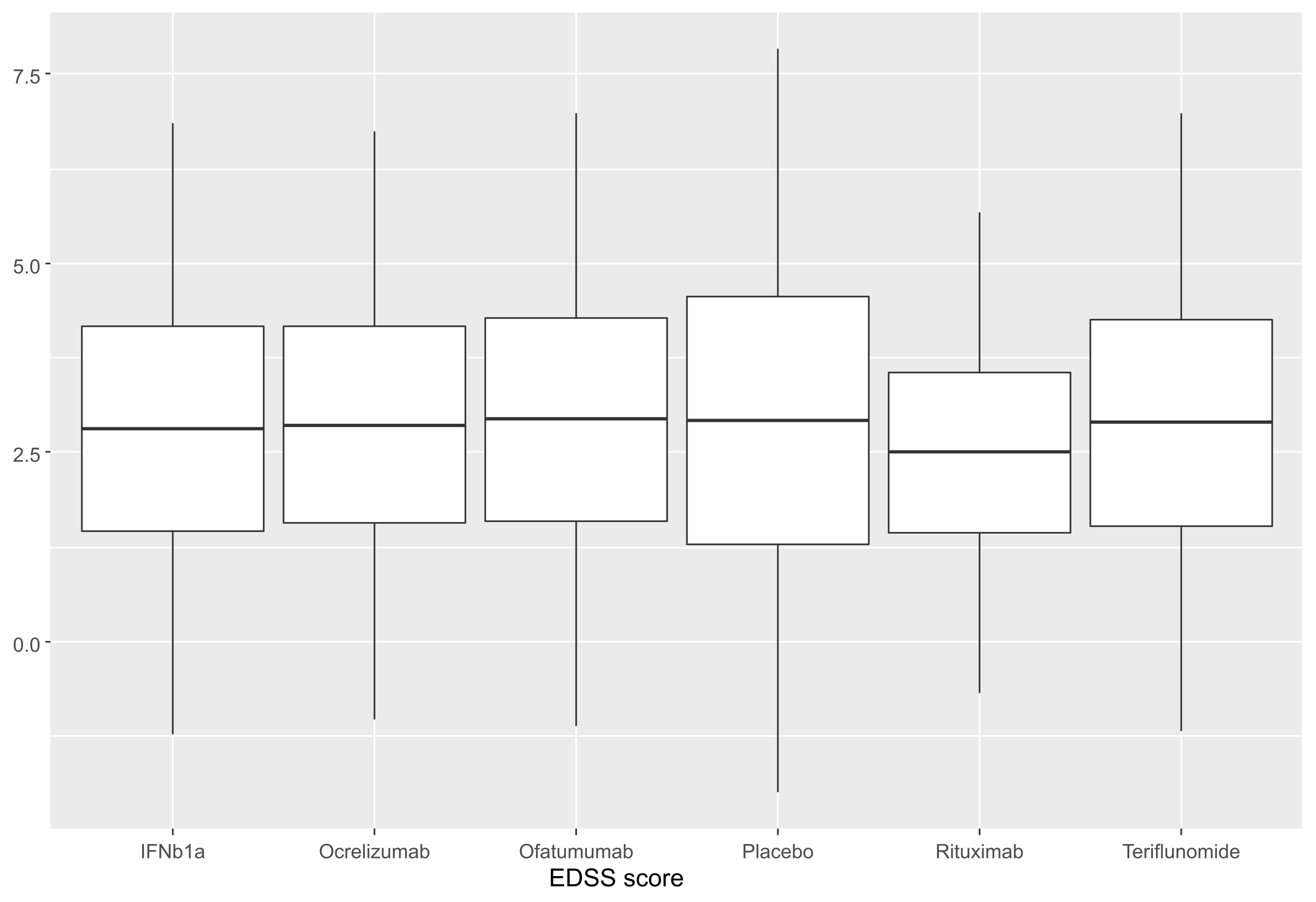
**

**6.5. Number of Relapses in the past year**

**
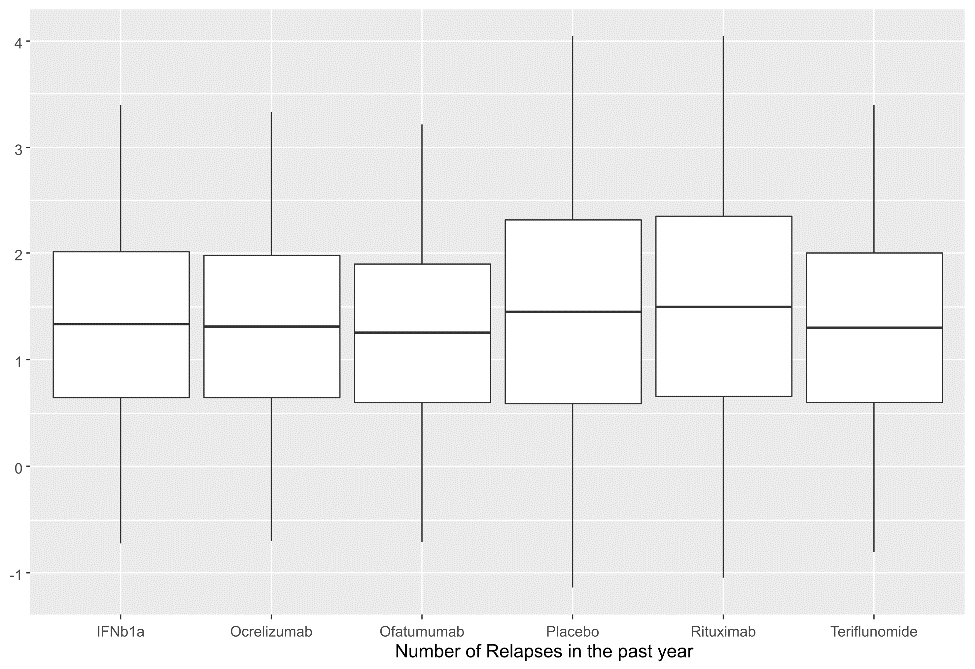
**

**Appendix 7:** Assessment of the inconsistency between studies


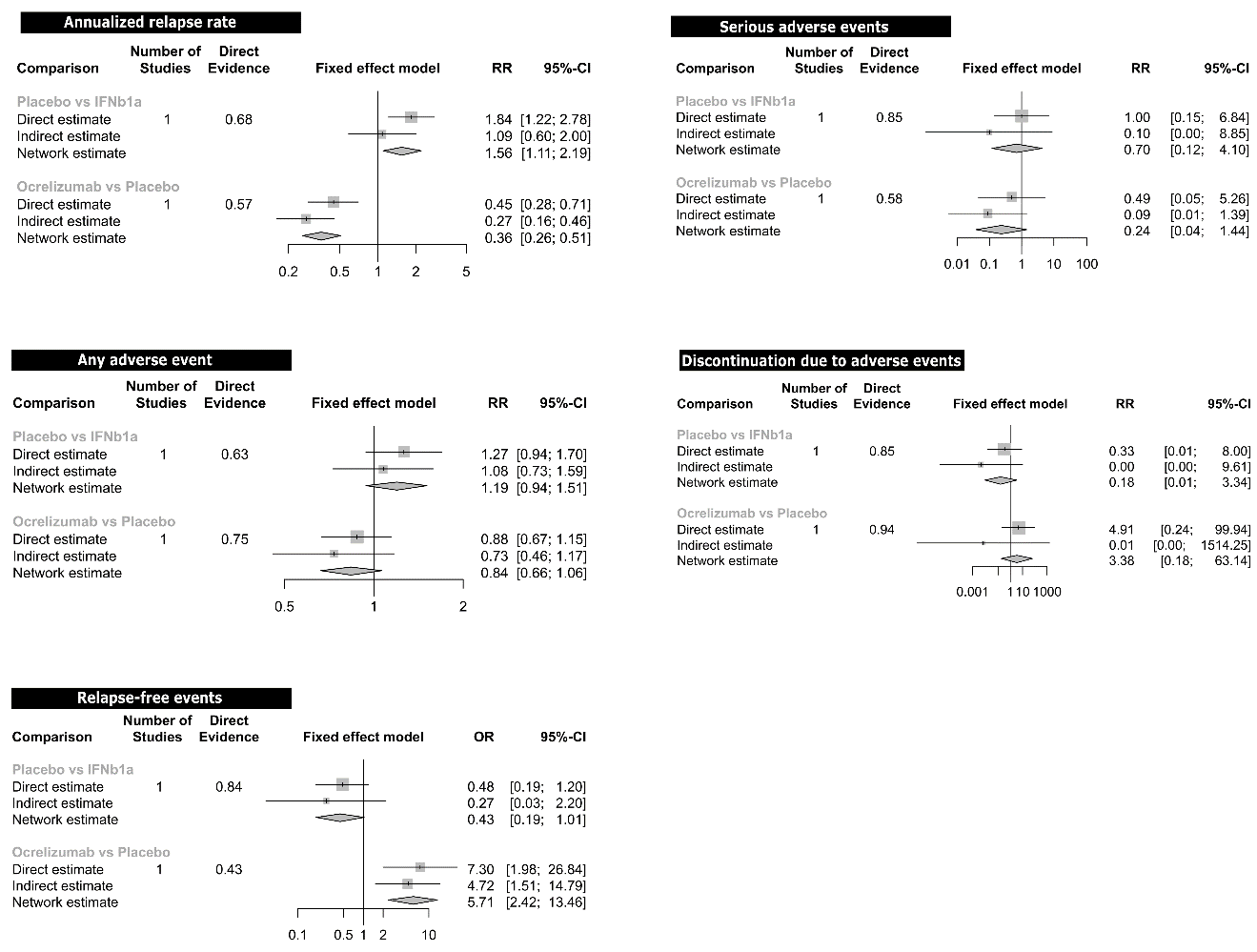


**Appendix 8:** Assessment of treatments rankings.

**8.1. P-scores of treatments ranking regarding the impact of different interventions on the risk of annualized relapse rates.**

| **Intervention** | **P-scores*** |
| --- | --- |
| Ocrelizumab | 0.9366 |
| Ofatumumab | 0.7207 |
| Rituximab | 0.5995 |
| IFNb1a | 0.4921 |
| Teriflunomide | 0.1273 |
| Placebo | 0.1239 |

*Treatment ranks close to 1 indicate a low risk.

**8.2. P-scores of treatments ranking regarding the impact of different interventions on the risk of experiencing serious adverse events.**

| **Intervention** | **P-scores*** |
| --- | --- |
| Ocrelizumab | 0.9280 |
| Rituximab | 0.5484 |
| Placebo | 0.5054 |
| Teriflunomide | 0.4013 |
| IFNb1a | 0.3684 |
| Ofatumumab | 0.2485 |

*Treatment ranks close to 1 indicate a low risk.

**8.3. P-scores of treatments ranking regarding the impact of different interventions on the odds of relapse-free events.**

| **Intervention** | **P-scores*** |
| --- | --- |
| Ocrelizumab | 0.9536 |
| Rituximab | 0.7132 |
| IFNb1a | 0.578 |
| Ofatumumab | 0.5067 |
| Placebo | 0.1871 |
| Teriflunomide | 0.0615 |

*Treatment ranks close to 1 indicate high odds of relapse-free events.

**8.4. P-scores of treatments ranking regarding the impact of different interventions on the risk of experiencing any adverse event.**

| **Intervention** | **P-scores*** |
| --- | --- |
| Ofatumumab | 0.6833 |
| IFNb1a | 0.6621 |
| Ocrelizumab | 0.6362 |
| Teriflunomide | 0.6161 |
| Rituximab | 0.277 |
| Placebo | 0.1253 |

*Treatment ranks close to 1 indicate a low risk.

**8.5. P-scores of treatments ranking regarding the impact of different interventions on the risk of treatment discontinuation due to adverse events.**

| **Intervention** | **P-scores*** |
| --- | --- |
| Rituximab | 0.7509 |
| Placebo | 0.689 |
| Teriflunomide | 0.5132 |
| Ocrelizumab | 0.4405 |
| Ofatumumab | 0.4292 |
| IFNb1a | 0.1772 |

*Treatment ranks close to 1 indicate a low risk.

**Appendix 9: Network meta-analysis of the secondary efficacy and safety outcomes**

**9.1.** No relapse at 24 weeks^¥^

| **Ocrelizumab** |  |  |  |  |  |
| --- | --- | --- | --- | --- | --- |
| 3.11 (0.77; 12.56) | **Ofatumumab** |  |  |  |  |
| 1.85 (0.51; 6.76) | 0.60 (0.14; 2.58) | **Rituximab** |  |  |  |
| **5.71 (2.42; 13.46)** | 1.84 (0.61; 5.52) | **3.08 (1.17; 8.10)** | **Placebo** |  |  |
| **8.52 (2.08; 34.94)** | **2.74 (2.23; 3.37)** | **4.59 (1.04; 20.20)** | 1.49 (0.49; 4.58) | **Teriflunomide** |  |
| **2.47 (2.00; 3.05)** | 0.79 (0.20; 3.19) | 1.33 (0.37; 4.82) | 0.43 (0.19; 1.01) | 0.29 (0.07; 1.18) | **IFNb1a** |

**9.2.** Any adverse event*

| **Ocrelizumab** |  |  |  |  |  |
| --- | --- | --- | --- | --- | --- |
| 1.03 (0.65; 1.65) | **Ofatumumab** |  |  |  |  |
| 0.85 (0.67; 1.08) | 0.82 (0.54; 1.23) | **Rituximab** |  |  |  |
| 0.84 (0.66; 1.06) | 0.81 (0.54; 1.22) | 0.99 (0.94; 1.05) | **Placebo** |  |  |
| 1.03 (0.64; 1.64) | 0.99 (0.95; 1.03) | 1.21 (0.80; 1.83) | 1.22 (0.81; 1.84) | **Teriflunomide** |  |
| 1.00 (0.96; 1.05) | 0.97 (0.61; 1.55) | 1.18 (0.93; 1.51) | 1.19 (0.94; 1.51) | 0.98 (0.61; 1.57) | **IFNb1a** |

**9.3.** Discontinuation due to adverse events*

| **Ocrelizumab** |  |  |  |  |  |
| --- | --- | --- | --- | --- | --- |
| 1.56 (0.03; 85.91) | **Ofatumumab** |  |  |  |  |
| 4.45 (0.15; 134.04) | 2.85 (0.11; 73.09) | **Rituximab** |  |  |  |
| 3.38 (0.18; 63.14) | 2.17 (0.14; 33.48) | 0.76 (0.13; 4.35) | **Placebo** |  |  |
| 1.70 (0.03; 95.34) | 1.09 (0.75; 1.59) | 0.38 (0.01; 10.04) | 0.50 (0.03; 7.98) | **Teriflunomide** |  |
| **0.60 (0.39; 0.93)** | 0.38 (0.01; 21.26) | 0.13 (0.00; 4.09) | 0.18 (0.01; 3.34) | 0.35 (0.01; 19.84) | **IFNb1a** |

^¥^ *Results are expressed as odds ratio (95%CI). * Results are expressed as RR (95% CrI). Comparisons between the column-defining and row-defining interventions should be read from left to right. The outcomes in bold and underline are statistically significant results.* *Secondary outcome; Treatment*
